# Cavitary Legionella Pneumonia in AIDS: When Intracellular Immunity Failure Leads to Rapid Intrapulmonary Cavitation

**DOI:** 10.1155/2021/6754094

**Published:** 2021-11-30

**Authors:** Richard Jesse Durrance, Alice Kyungsun Min, Marilyn Fabbri, Terrence McGarry

**Affiliations:** ^1^Division of Pulmonary and Critical Care, Icahn School of Medicine at Mount Sinai, Elmhurst Hospital Center, USA; ^2^Division of Infectious Disease, Department of Medicine, Icahn School of Medicine at Mount Sinai, NY, USA; ^3^Division of Infectious Disease, Icahn School of Medicine at Mount Sinai, Elmhurst Hospital Center, USA

## Abstract

*Introduction*. Legionella is a frequent cause of bacterial pneumonia in patients with AIDS. While multiple organisms have been associated with cavitary pneumonia in this population, Legionella has not. *Clinical Case*. A middle-aged woman with HIV-AIDS and severely depressed CD-4 count presented with one month of progressively worsening productive cough and dyspnea. Serial imaging showed focal consolidations which multiplied and cavitated over the subsequent days. Legionella urine antigen was positive, and appropriate treatment was continued for 3 weeks total. The patient recovered quickly, and follow-up imaging 8 weeks later showed near-resolution of all lesions. *Discussion*. Cavitary pneumonia secondary to Legionella has been seldom described, traditionally in the context of immunosuppressive therapy. Patients with AIDS and severely depressed CD4 counts have significantly compromised cell-mediated immunity. This case highlights the importance of consideration for legionellosis in rapidly progressing cavitary pneumonia, especially in patients with severely compromised cell-mediated immunity, including those with HIV-AIDS.

## 1. Introduction

Infection with HIV is associated with a significant increase in the incidence of bacterial pneumonia [1]. While S. pneumoniae, S. aureus, and H. influenza are the three principal causes of bacterial pneumonia in patients with AIDS, Legionella pneumophila is the fourth most common cause and has been reported to be up to 40 times more frequent in the AIDS population [1].

Cavitary lung disease in the HIV-infected patient carries a wide differential within both neoplastic and infectious etiologies, and in the patient with AIDS, opportunistic infections and certain malignancies are more likely [2]. Both bacteria (including mycobacteria) and fungi have been identified as the principal etiologic infectious agents of cavitary disease in three different retrospective case series evaluating cavitary lesions in patients with AIDS. However, in none of these series, covering three continents and 164 patients was Legionella identified as a causative agent [3–5]. On the contrary, Legionella has been described as the causative bacteria in a different class of immunosuppressed patients with cavitary lung lesions: recipients of kidney transplants [6].

We describe the case of a patient with HIV-AIDS and severely depressed CD-4+ cell count, presenting with rapidly evolving cavitary pulmonary lesions, of which Legionella pneumophila was identified as the underlying etiology.

## 2. Clinical Case

A 54-year-old woman diagnosed with HIV 30 years prior, and noncompliant with antiretroviral therapy (ART) over the past two years, presented to the hospital with persistent minimally productive cough and progressive dyspnea of 1-month evolution, associated with night sweats of 3-months evolution. She denied hemoptysis or unintentional weight loss, and review of systems was otherwise negative. She had a 20-year smoking history but had quit 2 weeks prior to presentation.

On exam, the patient had dyspnea and accessory muscle use when carrying on conversation and was visibly weak. However, the auscultatory chest exam was grossly unremarkable.

**Figure 1:**
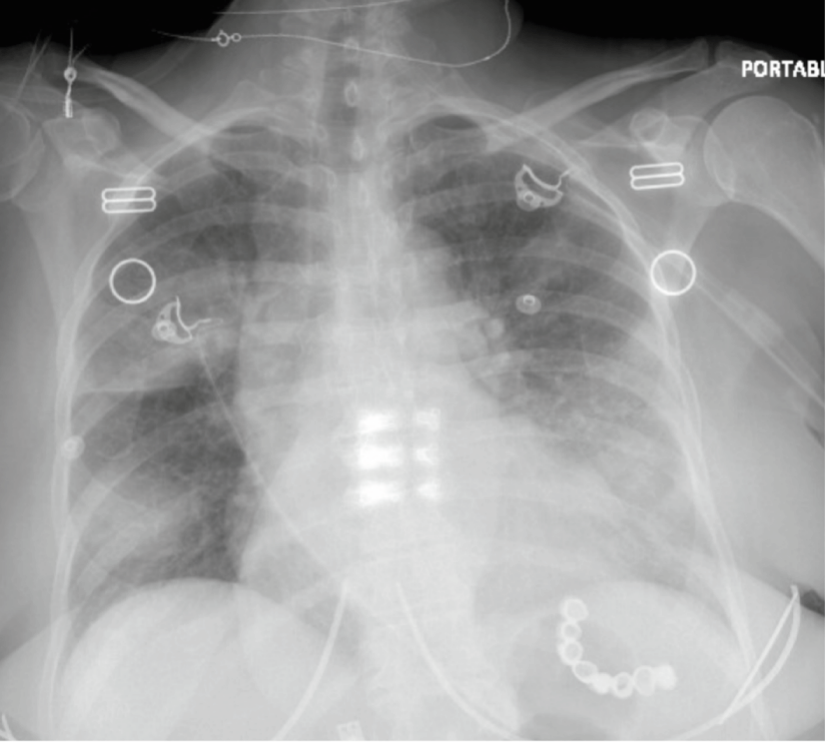
Portable posterior-anterior chest X-ray on admission showing multifocal ill-defined hazy opacities appreciated bilaterally.

**Figure 2:**
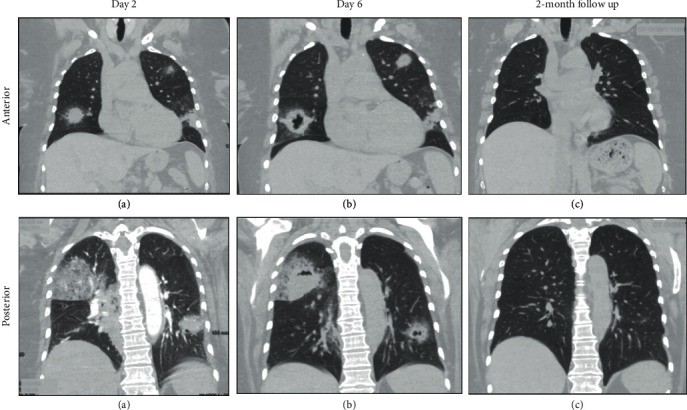
CT chest progression of pulmonary disease in anterior and posterior cuts of the coronal plane demonstrating rapidly cavitating disease. Chronology: (a) 2 days after admission; (b) 6 days after admission; (c) 2-month follow-up imaging.

Initial lab analysis showed a markedly decreased CD4 count (28/mm^3^), and mildly elevated LDH (318 U/L), but was otherwise unremarkable, and a COVID-19 swab was negative. The initial chest radiograph showed multifocal hazy ill-defined opacities bilaterally (Figure 1), and CT-chest imaging revealed bilateral nodular opacities in all lobes, with ground-glass halos, in both central and peripheral locations (Figure 2).

The patient was started on broad-spectrum antibiotics and admitted for further workup and monitoring. Initial microbiologic workup showed acid-fast-bacilli smears negative × 3. Her initial sputum culture was positive only for Enterococcus faecalis × 2, and a Legionella urine antigen was positive, for which the patient was suspected of having *Legionella* pneumonia and switched to targeted antibiotic coverage (amoxicillin-clavulanate × 1 week; azithromycin × 3 weeks total).

Repeat imaging showed progression of bilateral lung nodules with both growth and cavitation appreciated (Figure 2). Given the rapid evolution of disease, bronchoscopy with transbronchial biopsy was performed, revealing purulent discharge from the right upper lobe, and pathology showed acute and chronic inflammatory changes. No microorganisms were appreciated, DFA for *Legionella* was negative, and cultures, while sent 5 days after antibiotic initiation, were negative for *Legionella* and grew only *Enterococcus faecalis*, interpreted as a sputum colonizer.

Given the indeterminate nature of the histopathological findings and the positive Legionella antigen test, the patient was treated as presumptive Legionella pneumonia with a 6-week course of antibiotics. Clinical status significantly improved over the following days, and the patient was discharged within the week after being restarted on ART. On outpatient follow-up, the patient remained on ART and had complete resolution of her respiratory symptoms. Repeat imaging at 2-months postdischarge revealed near-complete resolution of consolidations and cavitations previously seen.

## 3. Discussion

Cavitary lesions in Legionella pneumonia have been described in infants [7, 8], in renal transplant patients [6], in adults with inflammatory/autoimmune diseases on immunosuppressive therapy [9], and in patients with primary immune disorders [10]. While immunosuppression appears to be an important component of the ability of Legionella to create extensive disease significant enough to cavitate, it is the suppression or depression of the acquired immune system and specifically defense against intracellular pathogens that is likely the key permissive factor. However, why different forms of cellular immunosuppression carry different susceptibilities to Legionella is unclear.

As a gram-negative bacterium ubiquitously present in the environment, Legionella only causes symptomatic infection when a sufficient concentration is able to enter the body. Innately, Legionella is taken up by macrophages. However, unlike most intracellular species (specifically mycobacterium amongst those that cause cavitary disease), Legionella rapidly replicates within the macrophage, a process that is actually facilitated by a robust humoral response to the bacteria, as antibodies promote their uptake by macrophages [11]. Therefore, when immunologic depression disproportionately compromises the cell-mediated pathway, susceptibility to severe Legionellosis markedly increases.

This is supported by a limited number of cases identified in the scientific literature in which Legionella has been identified as the causative agent of cavitary pneumonia. Cavitary lung lesions secondary to Legionella spp. were described in a series of renal transplant patients treated with azathioprine and prednisone [6]. While prednisone attenuates intercellular signaling, azathioprine diminishes acquired immune clonal expansion. This combination of immunosuppression likely allows an unchecked intramacrophage infection to progress to severe pneumonia with cavitary formation. In another case, a patient with hyper-IgE syndrome known to have markedly low serum IFN-𝛄 chronically developed cavitary pneumonia secondary to Legionella spp. after having received prednisone for 3 days [10]. The suppression of cell-mediated immunity again supports this as a strong factor allowing pulmonary legionellosis to progress to cavitary disease. These cases are similar in mechanism to that of a 31-year-old female ulcerative colitis patient who developed cavitary legionellosis after being treated with both prednisone and azathioprine [9].

Infants with cavitary pulmonary legionellosis appear to be a case apart however. As infants are born without a robust acquired immune system (both humoral and cell-mediated), targeting of intracellular organisms is more difficult. This likely results in facilitating the uptake of Legionella by macrophages without facilitating generation of an acquired cell-mediated response, thereby increasing the likelihood of clinically significant infection.

The overriding theme of the present case and past examples is a severe depression of cell-mediated immunity complemented by decreased inter- and intracellular signaling, thereby creating the opportunity for a relatively common respiratory pathogen to cause rapidly evolving cavitary pneumonia. It is worth noting that both DFA stains and cultures for *Legionella* on BYCE agar were negative. However, culture samples were not collected until 5 days after antibiotic initiation, thereby greatly decreasing its sensitivity. While these studies may have been able to identify other serotypes or species of *Legionella*, especially *Legionella micdadei* in which cavitary lesions are common [9], the positive urine antigen test early in the patient's course makes *Legionella pneumophila* serotype 1 the most likely diagnosis. In addition, while we recognize that *Enterococcus faecalis* was identified in the sputum and bronchial cultures, the fact that it is a frequent colonizer and rarely associated with pneumonia, much less cavitary disease, makes Legionella the far more likely etiology in this case. Furthermore, *E. faecalis* was found to be pan-resistant to all antibiotics used in this case.

## 4. Conclusion

While Legionella is a frequent cause of bacterial pneumonia, cavitary disease is exceedingly rare in the literature. In the circumstance of severely depressed cell-mediated immunity, including in patients with AIDS and very low CD4 counts, cavitary disease can occur, and Legionella must be considered in the diagnostic workup. Treatment must be promptly initiated, as delay can lead to a fulminant course, while prompt treatment initiation allows for lung recovery without sequelae.

## Data Availability

As this is a care report, no research data (i.e., database) was used or is available.
